# High resolution laboratory grating-based X-ray phase-contrast CT

**DOI:** 10.1038/s41598-018-33997-5

**Published:** 2018-10-26

**Authors:** Manuel Viermetz, Lorenz Birnbacher, Marian Willner, Klaus Achterhold, Franz Pfeiffer, Julia Herzen

**Affiliations:** 1 Chair of Biomedical Physics, Department of Physics and Munich School of BioEngineering, Technical University of Munich, 85748 Garching, Germany; 2 Department of Diagnostic and Interventional Radiology, Klinikum rechts der Isar, Technical University of Munich, 81675 München, Germany; 3 Institute for Advanced Study, Technical University of Munich, 85748 Garching, Germany

## Abstract

The conventional form of computed tomography using X-ray attenuation without any contrast agents is of limited use for the characterization of soft tissue in many fields of medical and biological studies. Grating-based phase-contrast computed tomography (gbPC-CT) is a promising alternative imaging method solving the low soft tissue contrast without the need of any contrast agent. While highly sensitive measurements are possible using conventional X-ray sources the spatial resolution does often not fulfill the requirements for specific imaging tasks, such as visualization of pathologies. The focus of this study is the increase in spatial resolution without loss of sensitivity. To overcome this limitation a super-resolution reconstruction based on sub-pixel shifts involving a deconvolution of the image data during each iteration is applied. In our study we achieve an effective pixel size of 28 μm with a conventional rotating anode tube and a photon-counting detector. We also demonstrate that the method can upgrade existing setups to measure tomographies with higher resolution. The results show the increase in resolution at high sensitivity and with the ability to make quantitative measurements. The combination of sparse sampling and statistical iterative reconstruction may be used to reduce the total measurement time. In conclusion, we present high-quality and high-resolution tomographic images of biological samples to demonstrate the experimental feasibility of super-resolution reconstruction.

## Introduction

To overcome the limited soft tissue contrast in conventional absorption-based imaging, several new X-ray phase-contrast imaging methods have been developed^1^. While most of these techniques are restricted to highly brilliant X-ray sources like synchrotron radiation sources, grating-based phase-contrast imaging^2^ has been successfully adapted to work with conventional X-ray sources^3^ and has become a promising candidate for medical diagnostics^4–8^ and industrial testing^9–11^.

In terms of contrast the differential phase X-ray imaging method allows for better differentiation of sample materials than conventional absorption imaging. However, not only contrast but also sufficient spatial resolution is necessary for optimal results in an imaging system.

**Figure 1:**
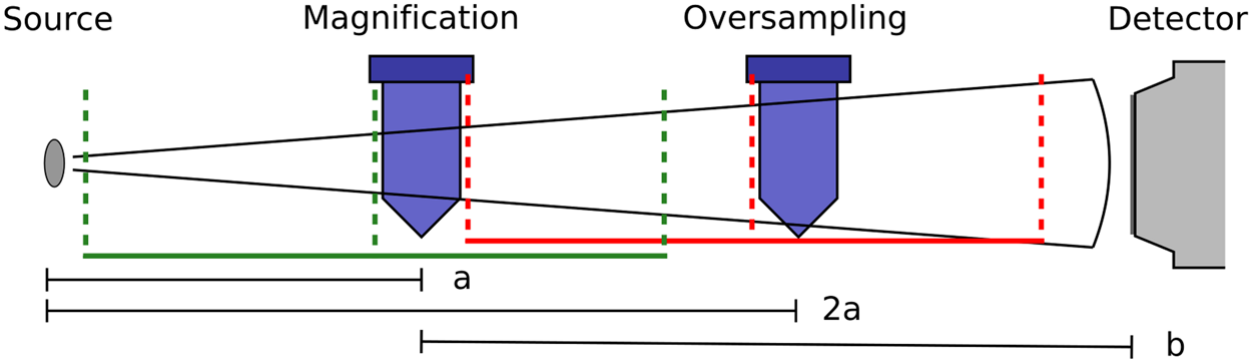
Illustration of the two setup geometries evaluated to increase the resolution. At the *magnification sample position* only geometric magnification is used for high resolution measurements. The resolution is limited by an extended source spot blurring the image. Super-resolution reconstruction is applied at the *oversampling sample position* at twice the distance from the source. The effective pixel size doubles and the influence of source blurring quarters for 2*a* = *b*. The images are sharp but resolution is low. For our grating-based phase-contrast setup *a* must be large enough for the gratings (in green for magnification case and red for oversampling) to fit in.

The most common method to achieve high resolution is to exploit the *geometric magnification* effect illustrated in Fig. 1. It is a standard approach for conventional absorption contrast imaging with limited soft tissue contrast^12,13^.

Phase-contrast imaging solves the soft tissue contrast problem; however, implemented as a Talbot Lau Interferometer the geometric flexibility of the setup is reduced. For highest sensitivity the sample must be positioned close to the central grating. This limits the minimum source-to-sample distance and thus maximum geometric magnification. Asymmetric setups allow for higher geometric magnification by shorter source-to-sample distance but require finer grating structures. Thus, the sensitivity is limited by the minimum fabrication dimensions^14–16^.

Common drawbacks in geometrical magnification are the significantly smaller field of view and the source magnification effect. The source magnification factor *M*_*s*_ = *b*/*a* (see Fig. 1) increases for higher geometric magnifications, which means that the projection is blurred by a magnified source intensity profile. This effect can be lessened by deconvolution of the image data.

In this study we demonstrate a strong increase in spatial resolution without loss in overall performance using super-resolution imaging, without any changes to the hardware. Two orthogonal sample stages are used to reposition the sample during oversampling while the detector stays at a fixed position. The setup in use has already been optimized for high sensitivity in a previous study^17^.

Multiple sub-pixel shifted images of the same scene are combined using an iterative algorithm to reconstruct the high-resolution image from the low-resolution images which contain no source blurring^18^.

## Experimental Setup

The presented setup consists of a high-flux rotating anode with a molybdenum target operating at 40 kVp with 0.3 mm focal spot size, a single-photon counting Eiger 1 M (Dectris Ltd.) detector and a Pilatus 100 K (Dectris Ltd.) detector used for imaging with a symmetric highly sensitive Talbot Lau interferometer^17,19^. The detectors utilize silicon sensors of 0.450 mm and 1 mm thickness, respectively.

The gold gratings have a period of 5.4 μm and are produced by the *Karlsruhe Institute of Technology* (*KIT*)^20,21^. The inter-grating distance is 50 cm, optimized for high spectral acceptance, leading to a visibility of approximately 36%^16^ for a spectrum filtered by the three gratings and a water tank of around 2 cm thickness. The sample is immersed in the water tank to suppress beam hardening artifacts.

The distance between source and detector is 2670 mm for all experiments, but different sample position as illustrated in Fig. 1 with *a* = 1030 mm are used. The sample is always positioned close to the central grating.

The number of tomographic angles is 800 and filtered back projection is used for the tomographic reconstruction of all depicted datasets^22,23^.

## Super-Resolution Image Reconstruction

**Figure 2:**
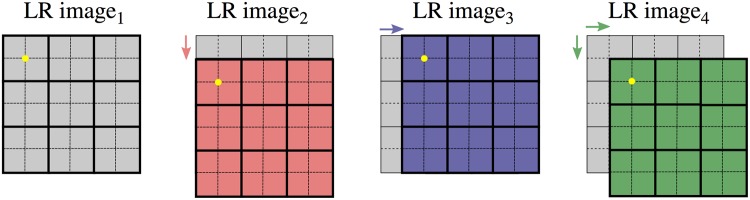
Illustration of the data acquisition procedure for 2 × 2 oversampling. In total four overlapping low resolution (LR) images are measured. This overlap can be used to reconstruct the HR image. The HR pixels are illustrated by dashed lines.

The super-resolution results presented in this study use 2 × 2 oversampling. This means that for each angular step of the measurement four images are taken as illustrated in Fig. 2.

In the first processing step these low resolution (LR) images pass the standard phase-contrast signal extraction procedure^24,25^. Then the information of the LR images is combined to obtain the high resolution (HR) image via a super-resolution reconstruction algorithm.

Many different approaches for super-resolution reconstruction algorithms have been proposed in recent decades. Relevant categories for computed tomography are, amongst others, the frequency-domain-based approach, interpolation-based approach, regularization-based approach, and learning-based approach^26,27^ that have been successfully applied to a wide range of practical use cases^28,29^. One of the most popular super resolution methods in medical image processing is *iterative back*-*projection* (*IBP*)^30,31^, which is applied in this study. In addition to being one of the most promising approaches, it has similarity to back projection used in tomography and thus may be directly implemented into the iterative reconstruction projectors at our group^18,32^.

The IBP algorithm in iteration step *n* can be expressed by

1 $${\hat{x}}^{n+1}={\hat{x}}^{n}+\sum _{k}\,({y}_{k}-{\hat{y}}_{k}^{n})\times {h}^{{\rm{BP}}}.$$

During the forward step four LR images ${\hat{y}}_{k}$ ($k=0\ldots 3$) are simulated from an initial HR image estimate $\hat{x}$ by multiplication with the system matrix *W*_*k*_. System properties such as the PSF can be implemented into this model for a better simulation result.

The resulting LR images are then compared to the measured LR images *y*_*k*_. During the backwards step, the error ${y}_{k}-{\hat{y}}_{k}$ is used to construct an improved HR image estimate via the back projection kernel *h*^*BP*^. This kernel contains information about how the different LR images contribute to the HR image estimate and may use prior knowledge of the scene. As IBP increases high frequency noise in the HR image, regularization can be added to limit the noise increase.

**Figure 3:**
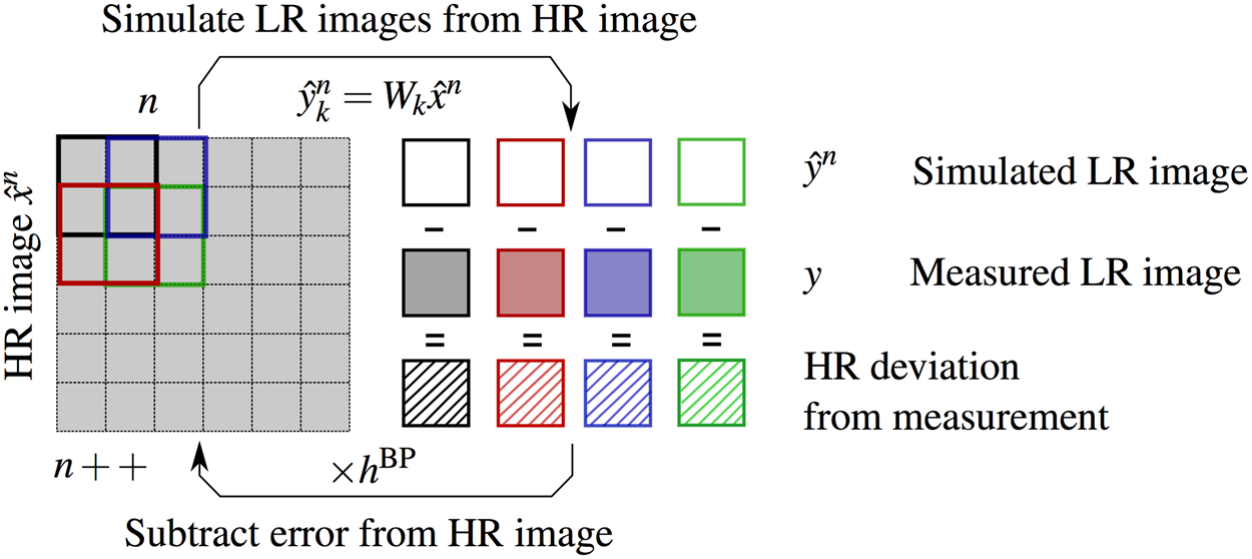
Super-resolution reconstruction using a recursive algorithm to minimize the error of the high resolution image. From the high resolution image the low resolution images are simulated. These simulated images are compared to the originally measured ones. The deviation is then subtracted from the high-resolution image during the back projection step.

The process is repeated iteratively until the quality of the HR image is sufficient or the noise variance exceeds a threshold. To ensure comparable datasets all reconstructions depicted in this study are optimized for the same noise variance. The concept of the described process is illustrated in Fig. 3.

Filtered back projection is used for tomographic reconstruction of the processed HR images. A common problem in the standard approach to tomographic reconstruction of conventional attenuation data is the use of filters with a large response to high frequency noise in the input data such as the Ram-Lak filter. As a consequence, noise generated from IBP is forwarded and amplified by the filter. The differential phase-contrast signal measured in this approach can only be filtered by a Hilbert filter, which has a low response to high frequency noise. Thus, this approach is robust to the increase in high frequency noise produced by IBP processing.

## Super-Resolution vs. Geometric Magnification

In this section we compare two phase-contrast measurements with the same effective pixel size of 28 μm; One using geometric magnification and the other applying oversampling as described in the previous section. For comparable results the interferometer setup is limited to 50 cm inter-grating distance and the noise variance is matched during reconstruction. Only the sample and interferometer position are changed while the total distance between source and detector stays constant. In Fig. 1 the two layouts for the magnification and the oversampling setup are illustrated in green and red, respectively.

The sensitivity of the interferometer is expected to be constant since the relevant parameters grating periods, inter-grating distance, visibility and noise stay constant for both measurements^17^.

For this measurement an Eiger 1 M detector with a 0.450 mm silicon sensor is used, leading to a long measurement time of approximately 18 hours for 800 projections with eleven phase-steps for each tomographic angle. In oversampling mode, four stepping curves are measured for every angular position while all other measurement parameters remain unchanged resulting in a total measurement time of 72 hours.

**Figure 4:**
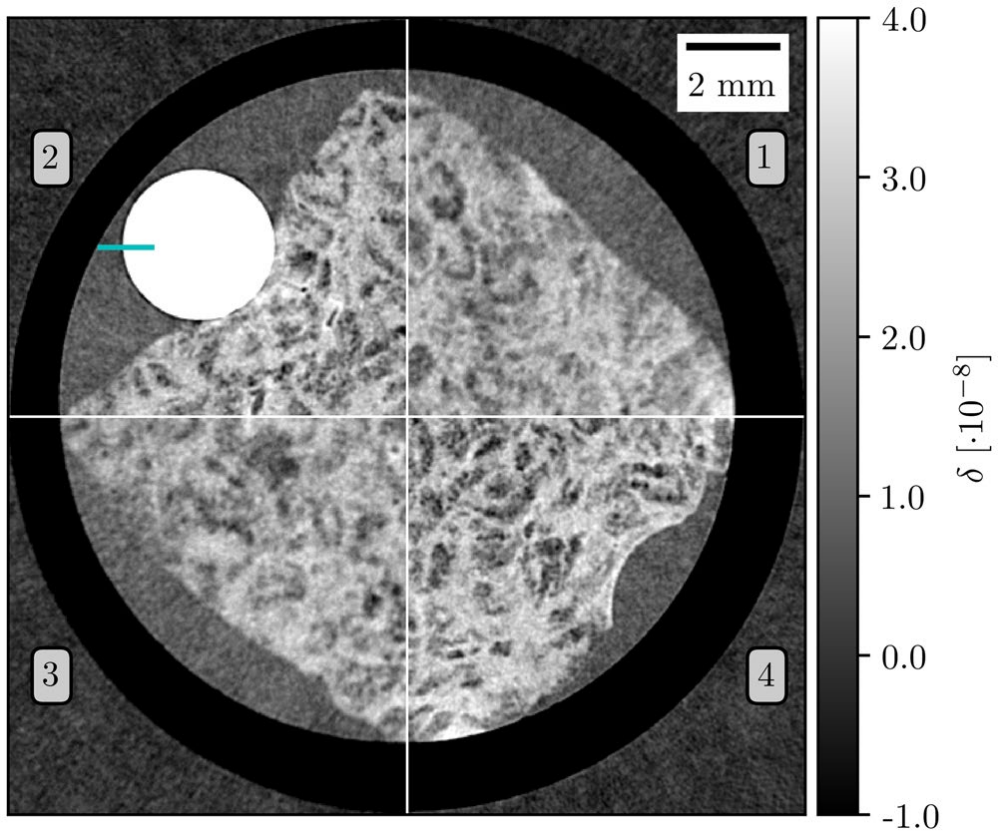
Tomographic reconstruction results of a liver cirrhosis sample using filtered back projection of differential phase-contrast measurements. Quadrants 1 and 3 show the measurement results of geometric magnification with an effective pixel size of 28 μm. In quadrants 2 and 4 oversampling is used to achieve the same effective pixel size. The increased sharpness in the super-resolution measurement is clearly visible while details are blurred away in the geometrical magnification by the extended source profile. A PMMA rod is used for calibration and ESF evaluation in Fig. 5.

To compensate for source blurring in the geometric magnification dataset, deconvolution is applied using the Lucy-Richardson algorithm. An exemplary slice is shown in Fig. 4. While the reconstruction from geometric magnification is blurred, the super-resolution dataset is sharply reconstructed without any blurring or artifacts. Apart from the quality improvement, the data is still quantitative after the super-resolution processing. A calibration based on the electron density of a PMMA tube (white) and the measured signal is used since it assures the compatibility between datasets with and without oversampling^33^.

**Figure 5:**
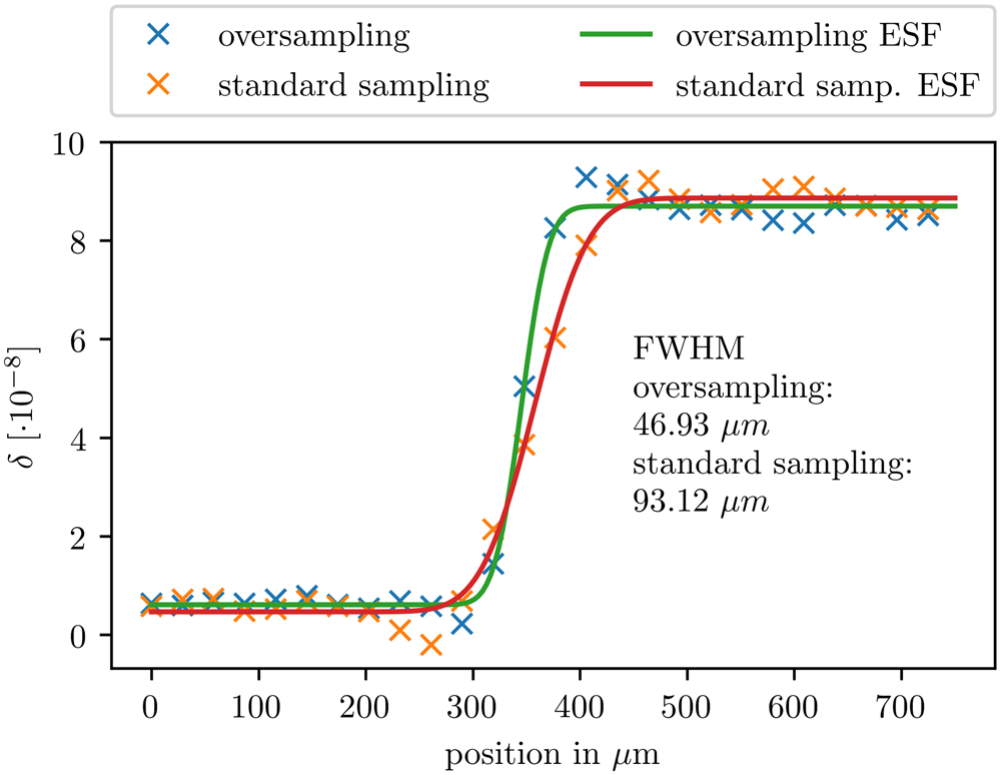
Super-resolution imaging delivers a sharper edge profile compared to the geometric magnification (standard sampling) approach. The resolution is increased by almost a factor of two. The ESF is measured from the sharp interface between sample fixation liquid and PMMA cylinder as highlighted in Fig. 4.

As a measure for the resolution enhancement, Fig. 5 shows the edge spread function (ESF) of the liquid-PMMA-interface. The dataset acquired via geometric magnification has a full-width-half-maximum (FWHM) of 93 μm. Oversampling leads to a significant improvement of the spatial resolution with a FWHM of only 47 μm.

## Super-Resolution vs. Standard Resolution

In this section we demonstrate application of the super-resolution imaging method at a high-sensitivity Talbot Lau interferometer in combination with a more efficient Pilatus 100 K detector. The inter-grating distance is extended to 85 cm, utilizing all available space between source and detector, maximizing the sensitivity. This detector has a 1 mm thick silicon sensor which has a better quantum efficiency than the previously used Eiger 1 M detector but has a larger pixel size of 172 μm. The effective pixel size of the setup is 83 μm. Eleven phase-steps with an exposure time of 10 s are used. The entire measurement takes five days because of the 2 × 2 oversampling steps for each projection. A standard measurement without oversampling would take 24 h. For standard reconstruction of the measurement only the first sub-image per angle is used. Optimization of measurement time is discussed in the next section.

**Figure 6:**
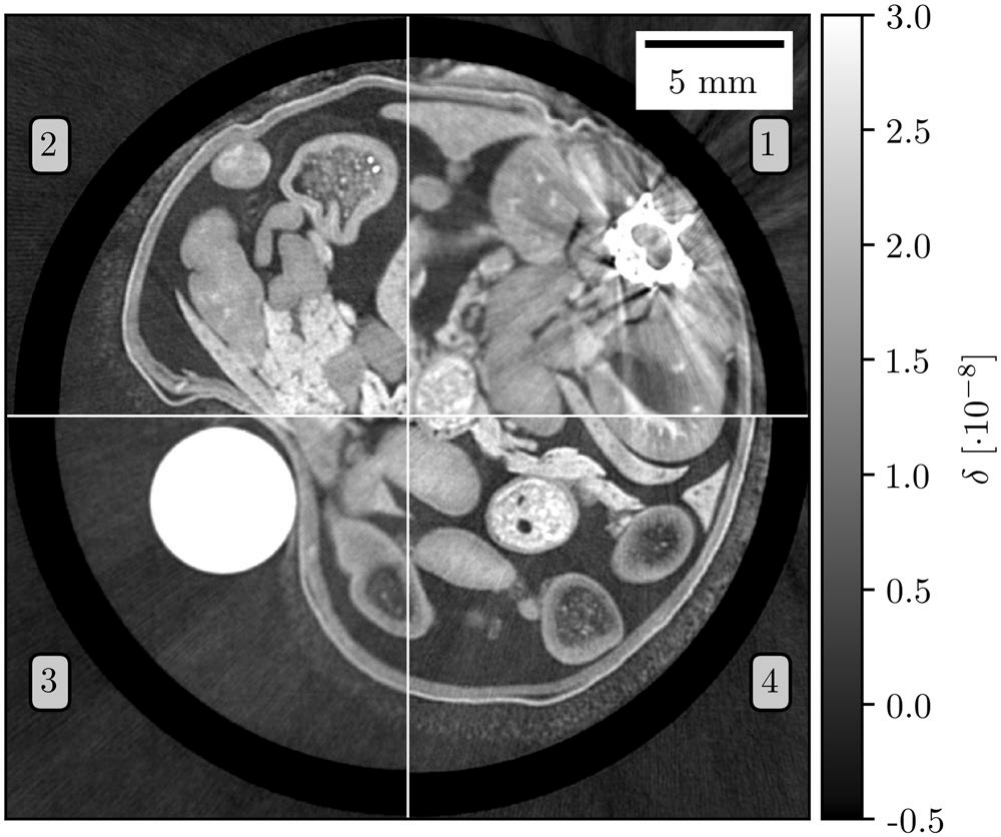
Tomographic reconstruction results of a formalin fixated mouse sample using filtered back projection of a differential phase-contrast measurement. In Quadrants 2 and 4 super-resolution reconstruction with an effective pixel size of 42 μm is shown. In comparison to the standard resolution reconstruction (effective pixel size 83 μm) in quadrants 1 and 3 fine features of the skin and organs can be distinguished and the result is generally sharper. Streak artifacts from beam-hardening caused by the spine are a common issue in imaging mice at low kVp.

The large field of view is demonstrated using a mouse sample fixated in formalin. The resulting tomographic reconstruction using filtered back projection on a voxel grid with 42 μm spacing is shown in Fig. 6. Quadrants 1 and 3 show the conventional reconstruction results with an effective pixel size of 83 μm. Here the lack of the detector’s spatial resolution leads to a slight blurring of the reconstruction because of the small voxel size. If a 83 μm grid is used this blurring is gone, but it is more difficult to compare the two datasets.

In the other quadrants the super-resolution reconstruction can retrieve more details and therefore provide a sharper tomographic slice.

This demonstrates that the super-resolution reconstruction approach is a powerful upgrade solution to increase the spatial resolution of an existing setup which is not limited by source blurring. No hardware modifications are necessary for CT setups that use a motorized x-y-stage to position the sample as it can also be utilized for the oversampling displacement.

## Application of Super-Resolution Reconstruction

Several studies report high resolution phase-contrast imaging results at synchrotron beamlines^34–38^. Good results can also be obtained using special liquid metal jet X-ray sources. These powerful laboratory micro-focus tubes with sufficient coherence are mainly used with propagation based phase-contrast imaging^39–42^. However, for biomedical studies high sample throughput, and thus availability, is important. The combination of a standard X-ray source with a super-resolution enhanced high-sensitivity Talbot Lau interferometer allows for future biomedical studies previously limited by spatial resolution.

The super-resolution approach allows for an immediate increase in resolution by simply adding more projection measurements without any hardware modifications. Especially for already optimized setups this method is useful to further increase the imaging performance for measurements where spatial resolution is essential.

An application of the presented method is illustrated using an unstained rat brain sample with a tumor. The specimen is measured with the objective to resolve even fine structures inside the cancerous tissue. It is important to emphasize that phase-contrast imaging grants high soft tissue contrast without any contrast agents. This makes phase-contrast imaging in combination with super-resolution imaging an universal tool for laboratory based biomedical studies.

**Figure 7:**
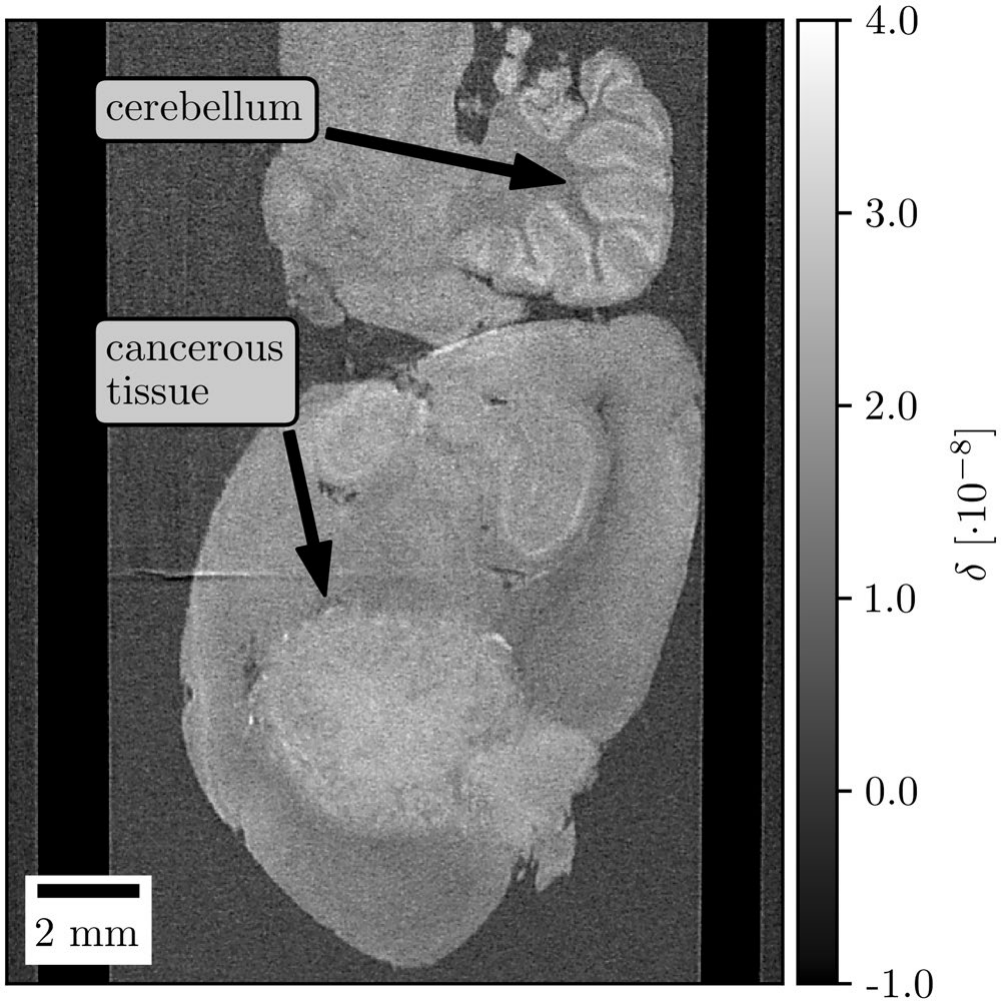
Exemplary tomographic slice of a rat brain sample with a tumor. The effective pixel size is 28 μm after the IBP super-resolution reconstruction of the oversampled data. The sample is represented sharply and tissue structures are visible.

In Fig. 7 the reconstructed dataset with an effective pixel size of 28 μm shows the rat brain sharp and with high contrast. The cerebellum can easily be identified and the cancerous tissue can be differentiated from the surrounding tissue.

This sample is measured with the same exposure time and number of angular steps as the mouse sample. To achieve the demanded high resolution the Eiger 1 M detector is used. The lower quantum efficiency of this detector is compensated by a thinner water tank which leads to an increase in X-ray flux on the detector.

The presented results clearly show that super-resolution imaging has the ability to provide superior image resolution at a grating-based phase-contrast setup compared to the standard approach. However, it is important to consider the longer measurement time. In terms of availability long measurement times at an X-ray tube are not as critical as they are at a synchrotron. Nevertheless, long measurements are not user-friendly and more likely to suffer from setup and sample instabilities. It is therefore important to evaluate how measurement time can be decreased and whether such a system could be set up with state of the art technology.

The detectors in use for this study are silicon-based photon counting detectors which have a comparably low quantum efficiency. With the development of new sensor materials, a significantly more efficient detector generation is now available to be utilized in modern X-ray imaging setups. Application of e.g. CdTe based detectors decreases the measurement time by more than a factor of two since their quantum efficiency is above 80%^43^. This is high compared to silicon sensors which have 80% quantum efficiency only for energies below 17.5 keV and rapidly decreasing efficiency for higher energies^44^. This shows that measurement time can be significantly reduced with state of the art X-ray detectors.

Further potential may be found in the field of grating fabrication. Improvements in grating quality and substrate transmittance increase the overall performance of the interferometer^20^. The absorption gratings used in this study are about 80 μm high, however currently structure heights of up to 250 μm are possible. With higher grating structures higher X-ray energies can be used leading to faster exposure times.

In addition, a combination of statistic iterative reconstruction with super-resolution reconstruction and sparse sampling might be useful to reduce data acquisition time.

This shows that there are several promising approaches to strongly reduce the measurement time in super-resolution mode.

## Conclusion

In this study we demonstrated the first super-resolution grating-based phase-contrast computed tomographies at a laboratory X-ray source with effective pixel sizes as small as 28 μm. Such highly resolved tomographies are by now only known from microCT setups which can not deliver the contrast and quantitative information of the sample^45^. The measurements verify that super-resolution reconstruction methods such as IBP can be directly applied on differential phase-contrast measurements. For grating-based phase-contrast imaging the presented super-resolution approach is preferable due to the intrinsic geometrical limitations of a Talbot Lau interferometer. Combining the high sensitivity of the setup with the high resolution approach allows for measurements at a laboratory X-ray source that are comparable to synchrotron measurements^34,35,37,38^.

However, the usually lower flux of the X-ray tube and the high number of overlapping stepping curves lead to much longer measurement times, which is the major drawback at laboratory setups. State of the art X-ray detectors with strongly increased quantum efficiency, gratings with higher aspect ratios and improved statistic iterative reconstruction with sparse sampling have the potential to make super-resolution imaging applicable at most setups. Depending on X-ray flux and sample size an optimized setup with state of the art components should be able to deliver measurement times below two days which are applicable in practice.

We show that the super-resolution approach is of practical use in biological, medical, and material science studies. While laboratory X-ray setups will probably never be able to compete with synchrotron sources in terms of acquisition time, they are significantly cheaper and more accessible. This may be a good tradeoff for the longer measurement time. Therefore the application of super-resolution grating-based phase-contrast imaging alleviates resolution limitations at high-sensitivity setups for studies that are not subject to time restrictions and profit from quantitative imaging results which could otherwise only be acquired at a synchrotron facility.
